# Treatment of bladder dysfunction with solifenacin: is there a risk of dementia or cognitive impairment?

**DOI:** 10.1590/1414-431X2021e11721

**Published:** 2022-01-25

**Authors:** L.P. Dantas, A.R.C.C. Forte, B.C. Lima, C.N.S. Sousa, E.C. Vasconcelos, P.H.C. Lessa, R.F. Vieira, M.C.A. Patrocínio, S.M.M. Vasconcelos

**Affiliations:** 1 Laboratório de Neuropsicofarmacologia, Núcleo de Pesquisa e Desenvolvimentos de Medicamentos, Departamento de Fisiologia e Farmacologia, Faculdade de Medicina, Universidade Federal do Ceará, Fortaleza, CE, Brasil; 2 Departamento de Urologia, Hospital Geral de Fortaleza, Fortaleza, CE, Brasil; 3 Universidade Federal do Amapá, Macapá, AP, Brasil; 4 Faculdade de Medicina, Centro Universitário Christus, Fortaleza, CE, Brasil; 5 Departamento de Anestesiologia, Instituto Dr. Jose Frota, Fortaleza, CE, Brasil

**Keywords:** Solifenacin, Dementia, Cognitive impairment, Anticholinergics, Bladder antimuscarinics

## Abstract

The use of bladder antimuscarinics is very common in the elderly. However, recent population-based studies that assessed the use of anticholinergics or bladder antimuscarinics showed an increased risk of dementia when these drugs were used for a prolonged period. Several of these population-based studies included patients who used solifenacin, which is a bladder antimuscarinic released in 2005 with the prospect of being a more selective antimuscarinic for M3 receptors (M3R), which could make it a safer drug when trying to avoid unwanted effects of older bladder antimuscarinics such as oxybutynin, especially with regard to changes in cognition. Since the various bladder antimuscarinics have distinct pharmacological characteristics, such as in the ability to penetrate the blood-brain barrier, in selectivity for muscarinic receptors, and in brain efflux mechanisms, their effects on the central nervous system (CNS) may vary. Solifenacin was the drug selected in this review, which aims to describe the results of several articles published in recent years reporting the effects of solifenacin on cognition or the risk of dementia development. Although preclinical studies show that solifenacin can also act on brain M1 receptors (M1R), short-term clinical studies have shown it to be safe for cognition. However, there are no long-term randomized studies that prove the safety of this drug for the CNS. Thus, until the safety of solifenacin has been established by long-term studies, it seems advisable to avoid prolonged use of this drug in elderly patients.

## Introduction

Recently, some population-based studies have been published associating the use of antimuscarinics in the elderly (including antimuscarinics for the treatment of voiding dysfunction) to an increased incidence of dementia and an increase in mortality ([Bibr B01]-[Bibr B02] [Bibr B03] [Bibr B04] [Bibr B05] 6). Anticholinergics have been frequently used in elderly patients, reaching 33% in the elderly population with dementia (7). As the incidence of overactive bladder (OAB) and urinary incontinence also increases with age, the coexistence of voiding dysfunctions and dementia as well as the high use of bladder antimuscarinics is common in elderly patients (8). In a study that evaluated 3.78 million elderly patients aged 65 years and older with dementia, it was reported that 1.02 million (26.9%) were taking potentially inappropriate anticholinergic medications, with the most frequently prescribed drugs being oxybutynin, solifenacin, paroxetine, tolterodine, promethazine, and cyclobenzaprine, showing a high prevalence of prescriptions for bladder antimuscarinics at this age (9). However, the evaluation of each of these antimuscarinics in relation to their effects on the central nervous system (CNS) must take into account each drug individually, since the different antimuscarinics have specific pharmacological characteristics that can interfere with the concentration and action of the drug in the CNS (10). Solifenacin, approved for clinical use in 2005, is one of the most used antimuscarinics for the treatment of bladder dysfunction (11-14) and has been associated with changes in cognition (1,2,6,15). Therefore, our aim was to review the literature on the effects of solifenacin on cognition or risk of dementia and to clarify whether this risk is also significant with a drug with greater selectivity for M3 receptors (M3R), such as solifenacin.

## Material and Methods

For this review article, we performed a search in PubMed and SCOPUS databases with the following keywords: ‘solifenacin’ or ‘anticholinergics’ or ‘antimuscarinics’ plus ‘dementia’ or ‘Alzheimer’ or ‘cognition’ or ‘cognitive impairment’. Preclinical studies and clinical trials on solifenacin were evaluated.

## Cholinergic mechanisms in dementia

Dementia is a heterogeneous clinical syndrome that can be classified into four main subtypes: Alzheimer’s disease (AD), vascular dementia, frontotemporal dementia, and Lewy body dementia, with Alzheimer’s disease being the most common (16). There are other causes of mild cognitive impairment (impaired cognition without decline in daily function) and dementia that may occur during life, such as vitamin deficiency (thiamine, vitamin B12), hypothyroidism, hydrocephalus with normal pressure, alcoholism, infections (e.g., HIV), intracranial masses, brain injury due to trauma (17). The importance of using some medications in the elderly with a potential deleterious effect on the central nervous system, such as anticholinergics, is also highlighted (18).

Acetylcholine is a neurotransmitter widely distributed in the CNS (Table S1) that binds to cerebral muscarinic receptors (M1R, M2R, M3R, M4R, M5R) and is essential for cognitive function. It is abundant in the hippocampus and has an important mediating role in the formation of semantic and episodic memory. The neurotransmitter has been associated with age-related dementias, as occurs in AD where the cholinergic system is greatly affected (19). Patients with early AD can present with atrophy of the cholinergic system in the basal forebrain, with a decrease in the number of cholinergic neurons and a lower transcription of the choline-acetyltransferase enzyme, which is necessary for the synthesis of acetylcholine in the brain (20-22). A decrease in the number of cholinergic receptors and acetylcholine binding in the hippocampus was also observed in AD patients (23).

Another factor supporting the importance of acetylcholine in preserving memory is that AD patients usually experience an improvement in memory at the start of treatment with acetylcholinesterase inhibitors such as donepezil, rivastigmine, and galantamine, although not permanent (24). When muscarinic receptors are blocked with scopolamine, memory encoding is also impaired (25).

## Factors influencing the action of bladder antimuscarinics in the CNS and specific pharmacological characteristics of solifenacin

Choosing the safest anticholinergic for the CNS involves the following variables: lowest ability to cross the blood-brain barrier (BBB), being a substrate for P-glycoprotein, and lowest affinity for muscarinic M1R, which are mostly related to cognitive impairment (26,27). P-glycoprotein is responsible for an efflux transport in the CNS, pumping molecules out and influencing the accumulation of drugs in the CNS (17). The BBB becomes more permissive in conditions such as aging, stroke, diabetes, trauma, multiple sclerosis, AD, and Parkinson’s disease (26,28). The BBB permeability is directly proportional to patient age due to the shrinkage of epithelial cells and opening of tight junctions (27). Hydrophobic, lipophilic, neutrally charged, and smaller molecules (<400 kDa) can penetrate into the CNS more easily (29). In elderly patients, there are also fewer muscarinic receptors in the central nervous system, which could increase the effects of bladder antimuscarinics in the brain (30). The pharmacokinetics of individual drugs can also change with age. For example, a study of multiple doses of solifenacin (5 or 10 mg) in elderly patients (65 to 80 years) showed that Cmax (maximum concentration) and AUC (area under the plasma concentration-time curve 0-24 h) levels were 16% and 20% higher, respectively, compared with younger volunteers (18 to 55 years). The t1/2 at the 5-mg dose increased from 59 h in younger men to 75 h in older men and from 53 to 68 h in women (31).

Solifenacin succinate is a butanedioic acid salt with a molecular weight of 480.5 kDa, slightly basic (pKa 8.5), highly lipophilic with an octanol:water distribution of 50:1 at pH 7.0, being 93% positively charged at pH 7.4 (31).

Solifenacin, such as darifenacin, is a tertiary amine larger than other antimuscarinic agents like oxybutinin, presenting a lower capacity to penetrate the BBB (32,33). However, solifenacin does not have an efflux mechanism like darifenacin, transported by P-glycoprotein, and trospium chloride, transported by a multidrug-resistance-associated protein (MRP), which favors its accumulation in the CNS (33).

Cognitive dysfunctions induced by antimuscarinic agents are mediated mainly by the M1R and M2R in the CNS (34). If M1R and M2R are blocked, there may be changes in cognitive function, such as learning and memory deficits (27). In this sense, once the BBB is crossed, solifenacin binds to all muscarinic receptors (Table S1) but exhibits a relatively higher affinity and specificity for the muscarinic M3R subtype (pK1 affinity 8.0) than for the M1R (pKi affinity 7.6) and M2R (pKi affinity 6.9) subtypes (35-37).

Thus, despite being more selective for M3R, solifenacin also binds to M1R and M2R (35-37) and does not have a cerebral efflux mechanism (33), which could favor its accumulation in the CNS and provide long-term deleterious effects, especially in the elderly, who have greater permeability of the BBB and fewer muscarinic receptors in the CNS (27,28).

The metabolism and elimination of solifenacin occurs mainly through non-renal mechanisms, and this drug is primarily metabolized in the liver with the production of 4 metabolites: M2 (N-oxide of solifenacin), M3 (4R-hydroxy-so-li-fe-na-cin), M4 (4R-hydroxy N-oxide of solifenacin), and M5 (N-glucuronide of solifenacin) (38). The M2, M4, and M5 metabolites are inactive, with no pharmacological activity (38). Although the M3 metabolite has an affinity for muscarinic receptors similar to solifenacin (greater affinity for M3R), it has very low plasma concentrations and low potency, and its clinical effect is not significant (29,31,38).

Two studies reported different findings regarding the possibility of solifenacin penetrating the BBB. Callegari et al. (39) reported that, of the antimuscarinics used in the treatment of OAB, oxybutynin, solifenacin, and tolterodine had the highest cerebral concentration. Suzuki et al. (40), on the other hand, reported that solifenacin was rarely observed in the brain of rats, which may indicate that it does not significantly penetrate the BBB. In this study, Wistar rats aged 8 weeks were used and solifenacin was administered intravenously 10 min before the acquisition trials. The fact that young rats were used rather than older or senescent animals in the period, which would be equivalent to the age group in humans that most commonly use solifenacin, may have influenced the passage of solifenacin through the BBB (27,28,41). Furthermore, the use of a single administration of solifenacin 10 min before the trial may not have been sufficient to cause adverse effects on memory, as in population-based studies these effects are more evident with chronic use of medications such as bladder antimuscarinics (1,2,5,15).

## Effects of solifenacin on the CNS

The influence of solifenacin and other antimuscarinic agents on muscarinic receptors in the brain has been measured *in vitro*. Jakobsen et al. (42) used a radio receptor bioassay to compare serum concentrations of antimuscarinics used in the treatment of OAB with brain anticholinergic activity. They used tolterodine, oxybutynin, solifenacin, darifenacin, and 5-hydroxy-methyl-tolterodine (5-HMT, the active metabolite of fesoterodine). Tolterodine and 5-HMT had the highest anticholinergic activity, followed by oxybutynin. Solifenacin had one of the lowest anticholinergic activity, surpassing only darifenacin. A study by Suzuki et al. (40), in which the effect of various antimuscarinic drugs on learning was tested by performing a passive avoidance test in rats, presented evidence that oxybutynin and propiverine affected learning in a dose-dependent manner. Darifenacin and solifenacin did not affect learning, according to their tests.

A study by Kobayashi et al. (43) shows that the inhibitory effects of solifenacin on Ca 2+ mobilization stimulated by carbacol are equivalent to those of oxybutynin in detrusor cells, but much weaker in submandibular gland cells, suggesting that solifenacin has pharmacological selectivity in the bladder over other tissues. Maruyama et al. (44) evaluated the RO50 values (intravenous dose for 50% muscarinic receptor occupancy in the brain) and the inhibitory potency of increases in intravesical pressure (ID50) in an *in vivo* study in rats with intravenous injections of oxybutynin, propiverine, tolterodine, and solifenacin, through quantitative autoradiographic study. Considering the dose ratio (RO50/ID50) as a reflection of the selectivity of the bladder antimuscarinic agent in the brain, it was observed that this ratio was higher for solifenacin (8.1-46.7), tolterodine (3.6-17.9), and propiverine (2.2-8.9) than for oxybutynin (1.4-3.4), showing that solifenacin has greater selectivity for the bladder. Table 1 summarizes these *in vitro* and animal studies.

**Table 1:** Animal and *in vitro* studies with solifenacin.

Reference	Type	Drugs	Duration of treatment	Results
Callegari et al., 2011 39	Male Sprague-Dawley rats	5-HMT, darifenacin, oxybutynin, solifenacin, tolterodine, trospium	Single dose of the compound *sc* 1 h before animals were euthanized	Brain penetration was significant for oxybutynin, solifenacin, and tolterodine
Jakobsen et al., 2011 42	*In vitro* (anticholinergic radio receptor bioassay)	5-HMT, darifenacin, oxybutynin, solifenacin, tolterodine	NA	Solifenacin and darifenacin exhibited the lowest anticholinergic activity compared to the other drugs tested
Suzuki et al., 2007 40	Male Wistar rats	Darifenacin, oxybutynin, propiverine, solifenacin, tolterodine	The drugs were administered *iv* 10 min before the acquisition trials	Solifenacin did not affect learning in the passive avoidance test
Maruyama et al., 2008 44	Male Sprague-Dawley rats	Darifenacin, oxybutynin, propiverine, solifenacin, tolterodine	Single dose of compound *iv* 40 min before animals were euthanized	Solifenacin had a greater selectivity for the bladder over the brain compared to the other antimuscarinics

5-HMT: 5-hydroxymethyl tolterodine (the active metabolite of fesoterodine); *sc*: subcutaneously; *iv:* intravenously; NA: not assessed or not available.

Despite the concern about the use of antimuscarinics in the elderly, there are clinical studies demonstrating the safety of solifenacin in relation to cognition. A randomized, double-blind, triple-crossover study by Wagg et al. (30) with 26 patients older than 75 years and with mild cognitive impairment, comparing solifenacin 5 mg once daily, oxybutynin 5 mg twice daily, or placebo, during three treatment periods of 21 days each, separated by 21-day washout periods, concluded that solifenacin had no detectable effect on cognition, whereas oxybutynin was associated with a statistically significant decrease in both power and continuity of attention. The main side effects were mild, such as dry mouth and dyspepsia. In another randomized study, Kosilov et al. (32) evaluated 262 male patients, aged between 52 and 79 years, diagnosed with benign prostatic hyperplasia and OAB, with a minimum score of 24 points on the Mini-mental State Examination (MMSE) scale. The patients were divided into three groups in which the same dosage of tamsulosin (0.4 mg), different dosages of solifenacin (10 and 20 mg), and placebo were applied. After 8 weeks of study, there was no statistically significant variation in cognitive changes. Similar results were found in another study by Kosilov et al. (45) with 312 women, aged 60-83 years, with urge urinary incontinence or mixed urinary incontinence, with at least 24 points on the MMSE scale, who were randomized to solifenacin 20 mg daily and trospium 60 mg daily, solifenacin 10 mg daily and trospium 30 mg daily, or placebo. After an 8-week treatment period, there was no increased risk of cognitive impairment. The study by Wesnes et al. (46) analyzed the risk of cognitive impairment in the elderly comparing the use of 10 mg of solifenacin, 10 mg of oxybutynin, and placebo, without evidence of cognitive impairment with the use of 10 mg of solifenacin compared to the placebo group. Oxybutynin, on the other hand, has been associated with impaired cognitive functions, particularly in impaired attention and sustained attention power, working memory, and alert self-assessment. Fifteen adverse effects occurred in 10 subjects taking oxybutynin and only 3 adverse effects occurred in 3 subjects taking solifenacin, with drowsiness as the only adverse effect. The VEGA study was another investigation of the cognitive effects of solifenacin in the elderly population. This was an observational study with 774 patients over 70 years of age and OAB, who were treated with solifenacin 5 or 10 mg daily. After 12 weeks of treatment, no difference was observed in cognitive function evaluated with the MMSE scale (47). The use of solifenacin in stroke patients was also evaluated. Park conducted a follow-up study of stroke patients presenting symptoms of urgency and urinary frequency. Sixty-six patients received solifenacin (5 or 10 mg) and an age- and sex-matched control group of 66 subjects received placebo for 2 months. No significant difference was reported between groups in cognitive function (48).

The results of these studies show that the use of solifenacin in a short-term treatment (2 to 4 months) is safe, even in the elderly population using high daily doses of solifenacin, such as 20 to 30 mg per day (see Table 2 for an overview).

**Table 2:** Short-term human studies with bladder antimuscarinics (solifenacin included).

Reference	Design	Subjects (n)	Patients features	Antimuscarinics	Duration of treatment	Results summary
Wesnes et al., 2009 46	Randomized, double-blind, placebo-controlled study	12 patients	Over 65 years of age	10 mg SOL, 10 mg OXY and PLA	3 crossover periods of single dose treatment separated by two 14-day washout periods	No evidence of cognitive impairment with the use of 10 mg solifenacin compared to the placebo group. Oxybutynin was associated with impaired cognitive functions (impaired attention and continued attention power, working memory and alert self-assessment)
Wagg et al., 2013 30	Randomized, double-blind, triple-crossover trial	26 patients	Patients over 75 years of age and with mild cognitive impairment	SOL 5 mg once daily, OXY 5 mg twice daily, or PLA	3 treatment periods of 21 days each, separated by 21-day washout periods	Solifenacin had no detectable effect on cognition while oxybutynin was associated with a statistically significant decrease in both power and continuity of attention
Kosilov et al., 2018 32	Randomized study 3 groups	262 patients	Male patients aged 52-79 years, diagnosed with BPH and OAB, with at least 24 points on the MMSEs	Same dosage of TAM (0.4 mg) and different dosages of SOL (10 and 20 mg) and PLA were applied: SOL 10 mg +TAM or SOL 20 mg + TAM or PLA + TAM	8 weeks	No statistically significant variation in relation to cognitive changes
Kosilov et al., 2018 45	Randomized study 3 groups	312 patients	Women, aged 60-83 years, with urge urinary incontinence or mixed urinary incontinence, with at least 24 points on MMSEs	SOL 20 mg/d and TRO 60 mg/d, SOL 10 mg/d and TRO 30 mg/d or placebo: SOL 20 mg/d or TRO 30 mg/d or PLA SOL 10 mg/d or TRO 30 mg/d or PLA	8 weeks	No increase in cognitive impairment risk
Park, 2013 48	Retrospective case-control study	66 patients with stroke 66 controls	Stroke patients presenting urinary urgency and frequency symptoms	SOL 5 or 10 mg/d or PLA	2 months of solifenacin use	Solifenacin treatment did not affect short-term cognitive performance (evaluated with MMSEs or CDR-SB) in stroke patients
Triantafylidis et al., 2018 57	Systematic review	4 studies	Dual use of cholinesterase inhibitors and urinary anticholinergics in older adults	Concomitant use of cholinesterase inhibitors and urinary anticholinergics	NA	Inconclusive: no changes in cognition in 3 studies evaluated. Only one study showing an improvement in cognition with high doses of donepezil and solifenacin
Hampel et al., 2017 47	Observational study	774 patients	Patients aged ≥70 years with OAB	SOL 5 or 10 mg/d	12 weeks	No relevant effect of solifenacin on cognitive function in the MMSEs was observed in this elderly population

BPH: benign prostatic hyperplasia; CDR-SB: Clinical Dementia Rating Sum of Boxes; MMSEs: Mini-mental State Examination scale; NA: not assessed or not available; OAB: overactive bladder; OXY: oxybutynin; PLA: placebo; SOL: solifenacin; TAM: tamsulosin; TRO: trospium; d: day.

Despite the demonstrated safety in short treatments, there are no long-term follow-up studies that specifically assess the chronic use of solifenacin and its effects on cognition. Studies investigating the long-term effects of anticholinergics on the CNS are restricted to grouping and evaluating drugs into categories such as bladder antimuscarinics, which does not take into account the molecular differences between each drug that may impact pharmacokinetics and action on the CNS. Coupland et al. (1) conducted a cohort nested case-control study from 2004 to 2016 with 58,769 case patients (diagnosed with dementia during follow-up) and 225,574 matched controls. They found that exposure to several types of strong anticholinergic drugs is associated with an increased risk of dementia. There was a significant increase in risk of dementia associated with bladder antimuscarinics, which was proportional to the total standardized daily doses (TSDDs), which reflects the level of patient exposure to the drug, with an adjusted odds ratio of 1.65 in the highest exposure category (>1095 TSDDs). The adjusted odds ratio (aOR) for TSDDs less than 90 was 1.19, for TSDDs 91-365 the aOR was 1.35, and for TSDDs 366-1095 the aOR was 1.65. This means that a shorter exposure to these antimuscarinics may not cause the same effects as with long-term use. In another nested case-control study using the UK’s Clinical Practice Research Datalink, Richardson et al. (49) evaluated the association between newly diagnosed dementia patients and previous use of anticholinergics 4 to 20 years before the diagnosis of dementia. A total of 40,770 cases and 283,933 control subjects, aged 65–99 years, were included in the study. They concluded that there was a significant association between incidence of dementia and prescription of antidepressants, antiparkinsonians, or urological drugs with an anticholinergic cognitive burden (ACB) score of 3. ACB 3 urological drugs (oxybutynin and tolterodine were 2 of the 5 most commonly prescribed anticholinergics in this study) prescribed 15-20 years before the diagnosis of dementia showed a significant association with the incidence of dementia with an odds ratio of 1.27. Although solifenacin is not mentioned in that study, it is important to remember that this drug is classified as ACB 3 due to its high anticholinergic activity (50).

Gray et al. (2) conducted a prospective cohort study in which they evaluated 3,434 patients 65 years of age and older without dementia at study entry. They were followed for at least 10 years. During this study, 797 participants (23%) developed dementia, and there was a relationship between cumulative use of anticholinergics and incidence of dementia. This ratio was proportional to the total standardized daily doses (TSDDs) of anticholinergics dispensed in the last 10 years, with an adjusted hazard ratio (aHR) of 1.54 (95% confidence interval (CI): 1.21–1.96) for risk of dementia incidence with cumulative anticholinergic use >1095 TSDDs (more than 3 years of use) and only an aHR of 0.92 of (95%CI: 0.74–1.16) with 1–90 TSDDs. The aHR for cumulative use of anticholinergics between 91–365 TSDDs was 1.19 (95%CI: 0.94–1.51) and the aHR for TSDDs 366–1095 was 1.23 (95%CI: 0.94–1.62). In this study, 19% of the patients used bladder antimuscarinics, which were associated with an increased risk of developing dementia. In a retrospective population-based case-control study evaluating 1 million people in Taiwan, the effects of bladder antimuscarinics on the development of dementia were examined. The study followed patients diagnosed with dementia (20,246) and a control group without dementia (40,394 patients) aged 55 years and older with a mean age of 77 years, between 2000 and 2013, and excluded patients that used antimuscarinics for less than 1 year. The bladder antimuscarinics evaluated in this study were oxybutynin, solifenacin, tolterodine, propiverine, trospium, darifenacin, and fesoterodine. Patients using these medications exhibited a 2.46-fold increased risk of dementia compared to non-users (95%CI: 2.22–2.73) (15). Given the predisposition of dementia events in diabetic patients who also have a higher incidence of OAB, Yang et al. (6) studied the association of anticholinergics used in the treatment of OAB with the incidence of dementia. In this cohort study, 10,938 patients using OAB anticholinergics (oxybutynin, solifenacin, or tolterodine) for more than 28 cumulative defined daily doses were compared with 594,733 patients not using these medications. At the end of the 6-year period, 7,774 patients had dementia. The rate of dementia events in this period was 3.9% in the oxybutynin group, 4.3% in the solifenacin group, 2.2% in the tolterodine group, and 1.2% in the control group (P<0.001). The adjusted HRs of users compared to non-users of anticholinergic drugs was 2.35 (95%CI: 1.96 to 2.81) for oxybutynin, 2.24 (95%CI: 1.85 to 2.73) for tolterodine, and 2.16 (95%CI: 1.81 to 2.58) for solifenacin, showing that the use of solifenacin in diabetic patients increases the relative risk for subsequent diagnosis of dementia, although this is lower compared to the other antimuscarinics evaluated.

These population-based studies showed an increased dementia risk in patients taking bladder antimuscarinics (including solifenacin) for a period longer than 1 year, or even for shorter periods in diabetic patients, and this should be taken into account when evaluating the benefit-risk of using these medications in the elderly population (see Supplementary Table S2). It is also important to consider the concomitant use of other anticholinergic drugs, keeping in mind the total cholinergic burden, that is, the cumulative effect of taking one or more medicines with anticholinergic properties (50). These studies did not mention sex-related differences in the incidence of dementia.

Polypharmacy, especially the use of potentially inappropriate medications, has been common in the elderly (51). Older adults are more susceptible to adverse drug reactions due to age-related changes in pharmacokinetics and pharmacodynamics and comorbidity from chronic conditions, such as cardiovascular diseases and psychological disorders (52-54). In a study of adults aged 65 years and older, poor health status was found to be associated with use of potentially inappropriate medications (55). The use of these potentially inappropriate medications is sometimes neglected even in patients diagnosed with dementia. This was shown in a longitudinal study with adults aged 65 years and older newly diagnosed with dementia, in which the use of these medications increased by 11% compared with the year of dementia diagnosis (56).

The use of bladder antimuscarinics in patients already diagnosed with dementia has raised concern. In a systematic review on concomitant use of cholinesterase inhibitors and urinary anticholinergics, Triantafylidis et al. (57) analyzed the cognitive and functional results and the prevalence of association of these drugs. The prevalence of dual therapy ranged from 1.2 to 40.5% and mixed results were found for cognitive and functional assessment with dual therapy, with 3 studies reporting no changes in cognition and one study reporting improvement in cognition with high doses of donepezil and solifenacin. Thus, due to these mixed results, this systematic review was inconclusive.

## Avoiding bladder antimuscarinics

Antimuscarinic agents, such as solifenacin, are the first-line pharmacological treatment for OAB. However, mirabegron, a beta-3 agonist, has recently emerged as an alternative treatment that can prevent adverse CNS side effects of bladder antimuscarinics, which may be even more significant in patients already taking other antimuscarinics, thus avoiding an increase in total cholinergic burden. Total cholinergic burden should always be evaluated in polymedicated elderly patients, as it is a strong predictor of cognitive and physical impairments in these patients (18,50,58,59).

Mirabegron was approved for clinical use in 2015 and has demonstrated efficacy and tolerability in the treatment of OAB. In 2016, Warren and colleagues published a review on mirabegron. It was concluded that the drug is effective in controlling OAB symptoms and parameters such as number of micturitions per 24 h, frequency of incontinence episodes, and mean volume voided per micturition after a 4-week treatment (60). Welk and McArthur (5) conducted a retrospective cohort study with data from 2010 to 2018 comparing the incidence of dementia in 47,324 OAB patients treated with antimuscarinic agents and 23,662 treated with mirabegron. The group treated with antimuscarinics had a higher incidence of dementia compared to mirabegron (HR=1.23, 95%CI: 1.12-1.35). Men and those younger than 75 years had a higher incidence of dementia induced by antimuscarinics compared to those taking mirabegron. There was no significant difference between classes of anticholinergics used (oxybutynin, tolterodine, solifenacin, darifenacin, fesoterodine, trospium), and the group treated with solifenacin had the same propensity to develop dementia as other groups treated with other anticholinergics.

## Discussion

If antimuscarinics are chosen as a treatment option, it is recommended that all patients taking antimuscarinics who are at risk for cognitive impairment undergo periodic assessment of cognitive abilities using the MMSE scale (61). Memory changes in elderly patients are generally not noticed or reported by the patients themselves, making cognitive reassessment of elderly patients using antimuscarinics essential (27,61,62). Another important consideration is the use of bladder antimuscarinics in some elderly or bedridden patients with cognitive impairment. In these patients, this treatment may not provide quality of life benefits because some of these patients are unaware of urinary loss and do not perceive the social impact of urinary incontinence, thus generally not compensating for the risk of using these drugs (63).

According to the articles described in this review, clinical studies that used solifenacin for a short period of up to 4 months showed no changes in cognition or increased incidence of dementia, showing that the use of this drug for a few months (up to 4 months) is safe, even in the elderly population using high daily doses of solifenacin, such as 20 to 30 mg per day.

However, population-based studies evaluating the use of bladder antimuscarinics or anticholinergics showed a statistically significant increase in the incidence of dementia when these medications were used for more than 1 year (>365 TSDDs). Shorter exposure to these antimuscarinics did not show the same risk of dementia as long-term use, except in diabetic patients who used more than 28 cumulative defined daily doses. The effects of using these drugs between 4 months and 1 year on cognition remain inconclusive.

Although several of these population-based studies included patients who used solifenacin, we are not aware of any specific long-term studies of solifenacin with regard to its effects on cognition or risk of dementia, which limits the conclusion of this review regarding the risk associated with prolonged use of this drug. Thus, until the safety of solifenacin is established by long-term studies, it seems advisable to avoid prolonged use of this drug in elderly patients.

## Supplementary Material

Click here to view [pdf].
